# Study on the Immunomodulation Effect of *Isodon japonicus* Extract via Splenocyte Function and NK Anti-Tumor Activity

**DOI:** 10.3390/ijms13044880

**Published:** 2012-04-18

**Authors:** Yu-Jin Hwang, Jaehyun Kim, Dong-Sik Park, Kyung-A Hwang

**Affiliations:** 1 Department of Agrofood Resources, National Academy of Agricultural Science, RDA, Suwon 441-853, Korea; E-Mails: yujinh21@skku.edu (Y.-J.H.); kjh2002@korea.kr (J.K.); dpark@korea.kr (D.-S.P.); 2 Department of Biotechnology & Bioengineering, Sungkyunkwan University, Suwon 440-746, Korea

**Keywords:** *Isodon japonicus*, splenocyte, natural killer cell, cytokine

## Abstract

Here we investigated the potential immune-enhancing activity of *Isodon japonicus* on murine splenocyte and natural-killer (NK) cells *in vitro*. The ethanol extract of *I. japonicus* significantly enhanced the proliferation of splenocyte and induced the significant enhancement of NK cells’ activity against tumor cells (YAC-1). In addition, *I. japonicus* increased the production of interferon (IFN)-γ and tumor necrosis factor (TNF)-α, suggesting that the increase in NK cell cytotoxicity could be due to the enhancement of the NK cell production of both cytokines. Taken together, *I. japonicus* extract inhibited the growth of human leukemia cells (K562) by 74%. Our observation indicated that the anti-tumor effects of *I. japonicus* may be attributed to its ability to serve as a stimulant of NK anti-tumor activity. In addition, our results support the development of functional food studies on *I. japonicus*.

## 1. Introduction

Natural Killer (NK) cells are important in the first response against viruses and tumors. The fact that NK cells function in innate immunity is crucial to combat viral infection and destroy cancer cells [1,2]. Recently, studies have suggested that NK cells have characteristics of both the innate and adaptive immune systems. NK cells exert direct cytotoxic activity against tumor targets and can regulate the adaptive immune response by cytokine production [3–5]. Low NK cell activity is associated with an increase in the risk of carcinogenesis. Therefore, the focus of recent cancer treatment that promotes NK cells is to develop drugs.

Currently, there is considerable scientific and commercial interest in the continuing discovery of immune-modulatory agents as a new anticancer measure from natural product sources [6–8]. Korean wild edible vegetables are a very interesting source that enhances paraimmunity, the non specific immunomodulation of granulocytes, macrophages, NK cells and complement functions [9,10]. Specially, *Isodon japonicus*, a perennial plant grown extensively in Korea and Japan, is utilized as an edible vegetable and has long been used as a traditional medicine in East Asia for the treatment of gastrointestinal disorder, tumor and inflammatory disease [11–13]. Although many biological activities of *I. japonicus* are known, the related biological function and mechanism are scientifically unproved. Additionally, studies relating to immune systems are very rare. Thus, here we focused on the immunomodulation of *I. japonicus* and investigated how *I. japonicus* extract affects immune cell growth, cytokine production and inhibition on human cancer cells.

## 2. Results and Discussion

### 2.1. Cell Proliferation

**Figure 1:**
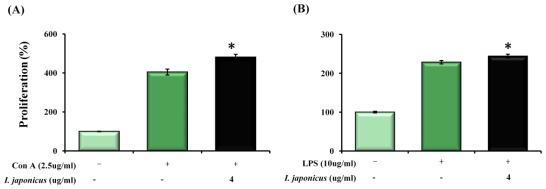
The proliferation effect of *Isodon japonicus* extract on T (**A**) and B lymphocytes (**B**). * *p* < 0.05.

The immune*-*modulatory effect of *I. japonicus* extract on murine splenocytes was investigated. The result of proliferation of T lymphocytes treated with mitogen (Con A) and I. japonicus extract showed an increase of 76.5% (*p* < 0.05) compared to the control as shown in Figure 1A. The proliferation of B lymphocytes stimulated with mitogen (LPS) and *I. japonicus* extract (4 μg/mL) resulted in a significant increase (15.6%) compared to the control as shown in Figure 1B. However, when treated with extracts on cells without mitogen, the proliferation of lymphocyte increased slightly but did not show significant increases (data not shown). Additionally, to confirm the T lymphocyte activation, we observed the expression of cell surface marker as CD25 and CD69 (Supplementary material). The results showed that the immune activation markers CD25 and CD69 significantly (*p* < 0.05) increased on the CD4^+^ and CD8^+^ T cell compared to the control of *I. japonicus* extract. An increase in the immune cell surface marker means that T lymphocyte is activated and that *I. japonicus* extract is able to increase the expression of these markers. This suggests that the extract has immune*-*modulatory potential. The results of our study showed that *I. japonicus* extract stimulated T, B lymphocytes and the stimulated immune cells may be attributed to inhibition of cancer cell growth.

### 2.2. NK Cells Activity

**Figure 2:**
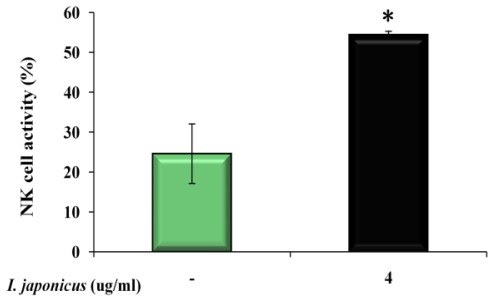
The natural killer cell activity of *Isodon japonicus* extract on murine splenocyte. * *p* < 0.05.

Since NK cells are known to be important effectors in suppressing tumor growth [14,15], we considered the activity of *I. japonicus* in enhancing NK activity and its relation to antitumor activity. We evaluated NK cell activity and the cytotoxic effect on target cells by co-culture with YAC-I as NK-sensitive cells. As shown in Figure 2, NK cells treated with *I. japonicus* represented over 30% cytotoxic activity compared to the control against YAC-I tumor cells at effectors with a target ratio of 10:1. Therefore, we suggest that, considering these results, increased NK cell activity could inhibit cancer cell growth as well as strengthen immune response and host defense.

### 2.3. Cytokine Production in Lymphocytes and NK Cells

**Figure 3:**
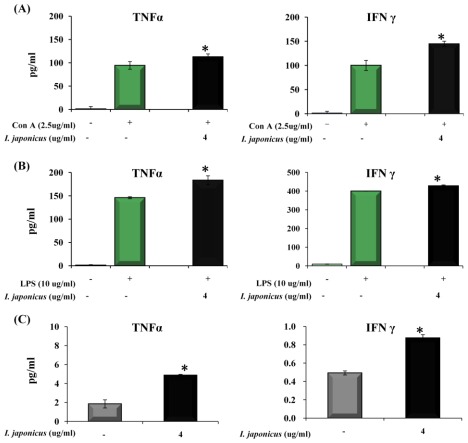
The effect of cytokines (TNFα and IFNγ) production by *Isodon japonicus* on T (**A**), B (**B**) lymphocytes and natural killer cell (**C**). * *p* < 0.05.

The effect of *I. japonicus* extract on the levels of immune-modulating cytokines as TNFα and IFNγ was analyzed (Figure 3A). The TNFα and IFNγ levels of T lymphocyte treated with *I. japonicus* extract increased by 18.4 and 44.4 pg/mL compared to that of the control (*p* < 0.05). Cytokine levels of B lymphocyte, TNFα and IFNγ were 37.3 and 27.8 pg/mL compared to the control, which was treated with mitogen (LPS) alone (Figure 3B). Furthermore, NK cells produced a very small amount of TNFα and IFNγ in comparison to lymphocytes. However, levels of TNFα and IFNγ increased by about 2 fold compared to the control (*p* < 0.05).

According to Biron *et al*., the secretion of cytokines in activated lymphocytes and NK cells play important roles in cancer cell suppression [16]. In particular, TNFα plays a role in activating T cells and rejecting tumor cells [17–19] and IFNγ inhibits tumor growth and metastasis by activating NK cells as well as T lymphocytes [20,21]. The results of our study were remarkably consistent with previous studies.

### 2.4. Cell Cytotoxicity Activity

**Figure 4:**
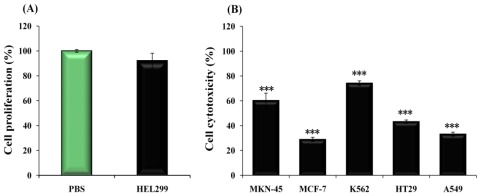
The effect of cell viability and cytotoxicity of *Isodon japonicus* extract on normal (**A**) and cancer cells (**B**). *** *p* < 0.001.

We next analyzed the cytotoxic effect of *I. japonicus* extract against five human cancer cell lines (stomach; MKN-45, breast; MCF-7, leukemia; K562, colon; HT29 and lung; A549). The *I. japonicus* extract had shown cytotoxic effect against all of the cancer cells (Figure 4B) and the most effectiveness on leukemia (K562) among all cancer cells, which was 74.1% (IC_50_ = 2.70 μg/mL) at 4 μg/mL. In addition, *I. japonicus* extract inhibited MKN-45, MCF-7, HT29 and A549 with IC_50_ values of 3.32, 6.942, 4.635 and 6.060 μg/mL, respectively, but the normal cell (HEL299) was unaffected with *I. japonicus* extract (Figure 4A). Our previous report [11] suggested that the polyphenol-rich wild edible vegetables could increase anticancer activity. However, interestingly, we observed that although *I. japonicus* extract had high polyphenol content, it showed relatively lower anticancer activity on HepG2 as human liver carcinoma cell and this trend was confirmed in several cancer cell lines (data not shown). These results indicated that the anticancer active compound of *I. japonicus* was not water-soluble polyphenol compounds, but that instead, other active compounds like phytochemical might contribute to its anticancer activity. Subsequently, we have isolated various presumed active compounds including kaurane diterpenoids from solvent extracts of *I. japonicas*, and further studies are underway to find the activity of immunomodulation and anticancer by kaurane diterpenoids, and a more in-depth analysis involving *in vivo* asthma models.

## 3. Experimental Section

### 3.1. Reagents

The reagent 3-(4,5-dimethylthiazol-2-yl)-2,5-diphenyltetrazolium bromide (MTT), dimethyl sulfoxide (DMSO), lipopolysaccharide (LPS), concanavalin A (Con A), were purchased from Sigma Chemical Co. (St. Louis, MO, USA). Dulbecco’s Modified Eagle’s Medium (DMEM), RPMI1640 medium, fetal bovine serum (FBS), phosphate-buffered saline (PBS), penicillin-streptomycin and trypsin-EDTA were obtained from Invitrogen Life Technologies Inc. (Carlsbad, CA, USA). Cytokine quantification kits were purchased from BD Pharmingen (Durham, NC, USA). Other reagents were used of analytical grade.

### 3.2. Sample Extract Preparation

The Korean wild edible vegetable, *I. japonicus*, was purchased from the Plant Extract Bank (Dae-jeon, Korea). The dried *I. japonicus* was milled into powder of 40 mesh particle size and extracted with 70% ethanol by stirring for 24 h at room temperature. The extract was filtered, and the residue was extracted in duplicate, under the same conditions. Subsequently, the filtrates were combined and evaporated under vacuum (EYELA N-1000, Tokyo Riakikai Co., Ltd., Tokyo, Japan) and then lyophilized with a Bondiro Lyophpride freeze dryer (Ilshine Lab Co., Ltd., Dongduchun, Korea) at −70 °C under reduced pressure (<20 Pa). The dry residue was stored at −20 °C. As preparation for further analysis, the dry residue was reconstituted with DMSO and diluted with PBS (pH 7.4) to the desired final concentration and filtered through a 0.45 μm syringe filter (Advanced MFS. Inc., Dublin, CA, USA) before use.

### 3.3. Cell and Cultures

HEL299 (KCLB NO.10137), A549 (KCLB NO.10185), HT29 (KCLB NO.30038), K562 (KCLB NO.10243), MCF-7 (KCLB NO.30022), MKN-45 (KCLB NO.80103) and YAC-I (KCLB NO.40160) cell lines were purchased from the Korean Cell Line Bank (Seoul, Korea). The cell lines were grown in RPMI 1640 medium or DMEM with 10% FBS and 1% penicillin–streptomycin, and incubated at 37 °C in 5% CO_2_.

### 3.4. Isolation of Splenocytes

The male C57BL/6J mice were obtained from SLC (Hamamatsu, Japan). The spleens were aseptically removed, and placed in RPMI 1640. Single cell suspensions were prepared passed through cell strainer (BD biosciences, Durham, NC, USA). And the red blood cells were lysed with Tris-NH_4_Cl. The splenocytes were cultured with sample and mitogen or without mitogen for specific cell differentiation to T and B lymphocytes [22].

### 3.5. Cell Proliferation and Cytotoxicity

The cell proliferation was measured by MTT assay [23]. Briefly, the isolated lymphocytes and cancer cells were seeded in 96-well culture plates. After 2 h, cells were treated 4 μg/mL of extract for 48 h. MTT solution (10 μL) was added and the cells were incubated for another 4 h. After removing the media, DMSO was added to each well to dissolve MTT-formazan product. The resulting absorbance was measured at 540 nm. Cell proliferation was expressed as percentage of viable cells of treated samples to control samples. All tests were performed in triplicate.

### 3.6. Natural Killer Cell Activity

Effector cell (isolated spleen cell) and target (YAC-I) cells were cultured at effector: target ratios of 10:1 in RPMI1640 containing 10% FBS and 1% penicillin-streptomycin. Cytotoxicity was assessed using MTT to measure cell viability [24]. The percent of specific cytotoxicity was calculated as follows:

$$\%\text{specific cytotoxicity}=[1-({\text{OD}}_{\text{effector}+\text{target}}-{\text{OD}}_{\text{effector}})/{\text{OD}}_{\text{target}}]\times 100$$

### 3.7. Determination of Cytokines

The supernatants of cultured spleen cells were harvested after 18 h. The concentration of each cytokine in the culture supernatants was determined by using ELISA commercially available from BD Pharmingen.

### 3.8. Statistic Analysis

Statistical analysis was performed with SPSS statistical software (version 17.0; SPSS Inc., Chicago, IL, USA, 2008). Descriptive statistics were used to calculate the mean and standard error of the mean (SEM). Student *t* test of variance was performed (*p* < 0.05).

## 4. Conclusions

In summary, we found that *I. japonicus* is critically involved in the T and B lymphocyte proliferation related to cell mediated and humoral immunity. The results were that the *I. japonicus* extract stimulated T, B lymphocytes and increased NK cell activity. Furthermore, the cytokines, the most important of the immunomodulation factors on immune cell stimulation, enhanced cell activity more than control groups. The IFNγ and TNFα helped to activate the T, B lymphocyte. Furthermore, more NK cells were produced than in control groups. These findings suggest that *I. japonicus* could inhibit cancer cell growth as well as generate humoral and cell mediated immune response and host defense through the activation of immune cell function. Thus, our results suggest that providing new research into the mmunomodulation effect of wild edible vegetable as japonicus extract as functional food materials has potential.

## Supplementary Information
