# Supplementary material for: Study on the Immunomodulation Effect of Isodon japonicus Extract via Splenocyte Function and NK Anti-Tumor Activity
Source: Int J Mol Sci. 2012 Apr 18;13(4):4880–8. doi: 10.3390/ijms13044880 (PMC3344252; doi:10.3390/ijms13044880)
Supplement: Supplementary file 1 [file ijms-13-04880-s001.pdf]

Supplementary Information

Figure S.1. The effect of *Isodon japonicus extract* on activation of cell surface marker in T lymphocyte.

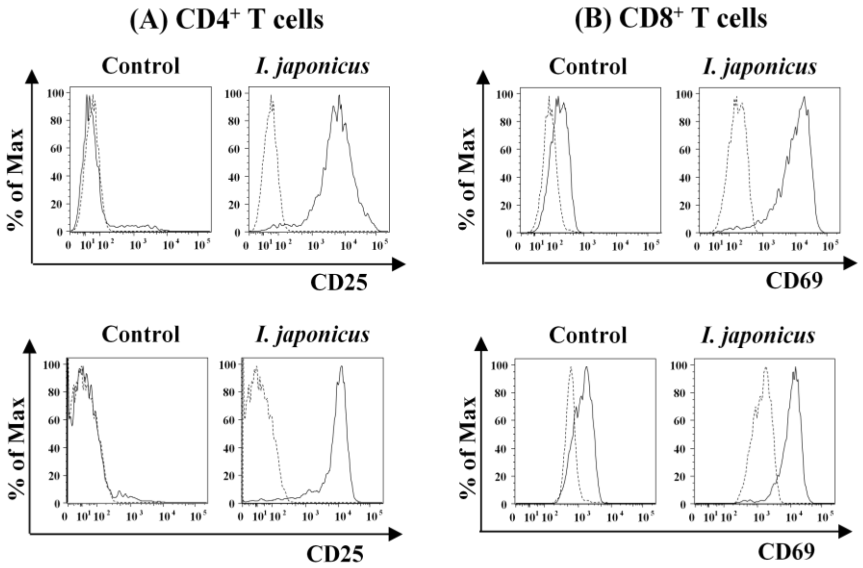

© 2012 by the authors; licensee MDPI, Basel, Switzerland. This article is an open access article distributed under the terms and conditions of the Creative Commons Attribution license (http://creativecommons.org/licenses/by/3.0/).
